# Protective Effects of Sesame Glycoproteins on Ultraviolet-Induced Skin Aging: In Vitro and In Vivo Studies

**DOI:** 10.3390/ph17101306

**Published:** 2024-09-30

**Authors:** Kyung Kyu Baik, Woo-Yong Song, Dong Keun Song, Jaehyeok Yun, Ji Hwan Jang, Jae Young Oh, Mi-Jin Lee, Eunjandi Go, Kyong Jin Lee, Eunmiri Roh, Jong-Eun Kim

**Affiliations:** 1 Department of Food Science and Technology, Korea National University of Transportation, Jeungpyeong 27909, Republic of Korea; bkk770@naver.com (K.K.B.); ehdrms0210@naver.com (D.K.S.); qweasd126@naver.com (J.Y.); c91084@naver.com (J.H.J.); jaeyong605@naver.com (J.Y.O.); 2 CNABIOTECH Co., Ltd., Cheongju-si 28106, Republic of Korea; sjousong@naver.com (W.-Y.S.); shuduc@hanmail.net (M.-J.L.); kejd9686@gmail.com (E.G.); jincrow@daum.net (K.J.L.); 3 Department of Cosmetic Science, Kwangju Women’s University, Gwangju 62396, Republic of Korea; roheunmiri@kwu.ac.kr

**Keywords:** sesame glycoproteins, ultraviolet, skin, aging

## Abstract

Background/Objectives: Ultraviolet (UV) radiation is a primary factor in skin photoaging, leading to wrinkles, reduced elasticity, and pigmentation changes due to damage to cellular DNA, proteins, and lipids. Glycoproteins from sesame cake (SPE) have potential protective effects against UV-induced skin aging. This study investigated the anti-photoaging effects of SPE on UV-induced damage in human keratinocyte HaCaT cells and SKH-1 hairless mice. Methods: SPE was evaluated for its ability to mitigate UV-induced damage in HaCaT cells by assessing MMP-1 protein and mRNA expression levels, as well as the activity of transcription factors AP-1 and NF-κB. The phosphorylation of AKT and MAPK pathways was also analyzed. In vivo, SKH-1 hairless mice were exposed to UV radiation, and the effects of SPE on wrinkle formation and skin structure were assessed by measuring wrinkle length, area, and volume. Results: SPE significantly inhibited UV-induced MMP-1 protein and mRNA expression in HaCaT cells, indicating suppression of AP-1 and NF-κB transcription factors involved in MMP-1 production. Additionally, SPE reduced UV-induced phosphorylation of AKT and MAPK pathways. In SKH-1 hairless mice, SPE treatment led to significant reductions in wrinkle length, area, and volume, preserving skin structure in UV-exposed mice. Conclusions: The findings demonstrate that SPE has protective effects against UV-induced photoaging by inhibiting key molecular pathways associated with skin aging. SPE shows promise as a natural anti-photoaging agent, providing a foundation for future skincare product development. Further studies are warranted to explore the molecular mechanisms in detail and to validate these effects through clinical trials.

## 1. Introduction

Exposure to ultraviolet (UV) radiation is a pivotal environmental factor that causes skin aging, a phenomenon known as photoaging [1]. Photoaging primarily results from the deleterious effects of UV radiation on cellular DNA, proteins, and lipids in the skin and causes wrinkles, diminished elasticity, and alterations in pigmentation [2]. In this context, the exploration and development of natural compounds to mitigate UV-induced skin damage has surged to the forefront of dermatological science [3]. Derived from a diverse array of sources, including plants, marine organisms, and microorganisms, these natural compounds are known for their antioxidant, anti-inflammatory, and moisturizing properties [2]. Strategic formulation of skincare products that incorporate these bioactive compounds is a promising avenue for bolstering skin defense mechanisms against UV radiation, thereby attenuating the characteristics of photoaging markers [3].

At the molecular level, UV exposure activates several signaling pathways, including the mitogen-activated protein kinase (MAPK) and phosphoinositide 3-kinase/AKT (PI3K/AKT) pathways [4]. These pathways regulate the expression of matrix metalloproteinases (MMPs), particularly MMP-1, which is responsible for collagen degradation [5]. The transcription factors AP-1 and NF-κB are key regulators of MMP-1 expression, and their activation is stimulated by UV-induced ROS [2]. In addition to triggering collagen breakdown, UV exposure also induces inflammation and cell death, further exacerbating skin aging [6]. Targeting these molecular pathways is crucial in preventing or mitigating UV-induced photoaging [5,7].

Sesame seeds are recognized as nutritional powerhouses, not only for their culinary versatility but also for their extensive health benefits [8]. These seeds are rich in proteins, unsaturated fats, antioxidants, and phytochemicals that contribute a wide array of health-promoting properties [9]. Key components such as sesamin and sesamolin, which are unique lignans found in sesame, exhibit potent antioxidant and anti-inflammatory properties [10]. These bioactive compounds exert protective effects against various chronic conditions, including cardiovascular diseases and diabetes [9]. In addition, the high dietary fiber content of sesame seeds supports digestive health and aids in glycemic control, underscoring their systemic health benefits [8].

Sesame seeds have shown great promise in promoting skin health owing to their rich nutrient content [8]. The antioxidant properties of sesamin help to fight oxidative stress in skin cells, which is a major factor in aging and skin disorders [11]. Sesame oil, which is high in omega-6 fatty acids, improves the skin barrier function, hydration, and recovery, suggesting that the consumption of sesame seeds can improve skin health [9]. Additionally, studies have shown that sesame oil can act as a natural sunscreen to protect against UV radiation and reduce sunburns and signs of aging from sun exposure [12].

Plant-derived glycoproteins offer various scientific benefits for improving skin health. First, the glycoproteins extracted from *Gardenia jasminoides* and *Cudrania tricuspidata* exhibited strong antioxidant and anti-inflammatory properties. These effects help reduce oxidative stress and inflammation in skin cells, thereby preventing premature aging and promoting healing [13]. Additionally, glycoproteins from sea cucumbers inhibit tyrosinase and elastase activities, which contribute to skin whitening and wrinkle reduction, making them valuable cosmetic ingredients [14]. Moreover, glycoproteins from *Dioscorea batatas* significantly enhance wound healing by promoting cell migration and epithelial tissue repair, thereby improving the overall skin health [15]. Furthermore, glycoproteins from *Rubus chingii* show antiaging effects by inhibiting oxidative stress and enhancing the expression of antiaging genes, suggesting systemic benefits that translate into skin health [16]. Finally, glycoproteins from *Withania somnifera* inhibit hyaluronidase activity, maintain extracellular matrix integrity, and contribute to overall skin resilience [17].

Utilizing sesame seed cake, a by-product of sesame oil extraction, for glycoprotein extraction is a sustainable and economically viable approach [18]. This strategy not only adds value to waste products but also aligns with the increasing emphasis on sustainability in the skincare industry [19]. By converting this by-product into a novel skincare ingredient, we can enhance the economic viability of sesame seed processing while advancing the development of effective and natural skincare products [20]. This approach promises benefits for both the agricultural and consumer markets, optimizing resources and fostering innovation in dermatological science. Sesame seed cake is rich in proteins and bioactive compounds, such as lignans and phenolics, which have demonstrated significant antioxidant and anti-inflammatory activities [21]. The high protein content in sesame seed cake can be leveraged to create high-value protein concentrates, adding further value to this byproduct [20]. For instance, sesaminol diglucoside isolated from sesame cake has strong antioxidant, anticollagenase, and antihyaluronidase activities, making it a promising candidate for skincare applications [18].

This study focused on the extraction and evaluation of glycoproteins from sesame seed byproducts, particularly in the context of UV-induced skin aging, using HaCaT cells and animal models. The primary goal of this study was to investigate the potential skin health benefits of these glycoproteins and introduce an innovative approach to combat photoaging. By understanding the protective capabilities of these natural compounds against UV damage, we aimed to contribute to the development of advanced skincare solutions that harness the innate protective potential of sesame seeds. This sustainable approach not only supports environmental conservation but also drives economic benefits for the agricultural and skincare industries.

## 2. Results

### 2.1. Characterization of Sesame Protein and Its Effect on HaCaT Cell Viability

**Figure 1:**
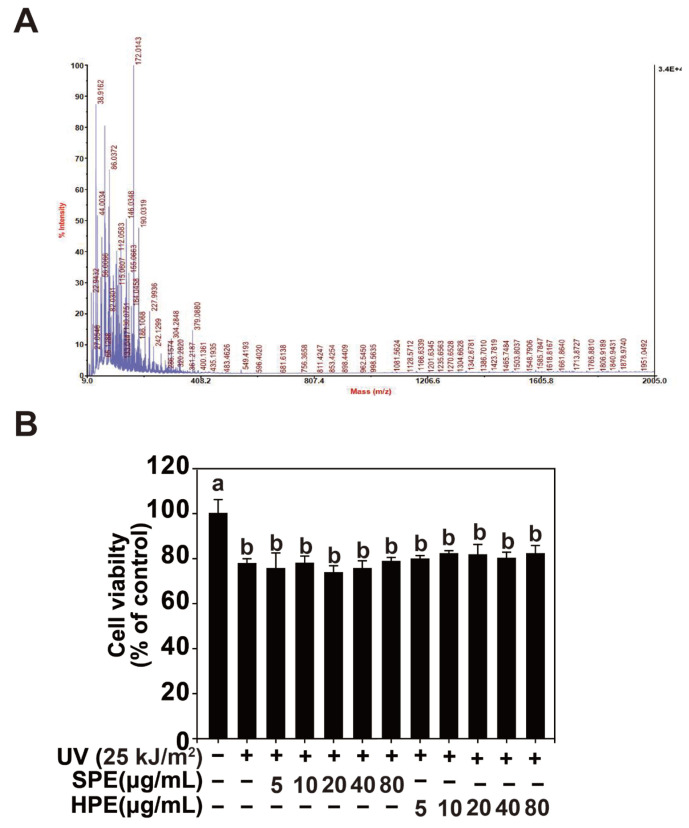
Molecular weight distribution and cell viability of sesame glycoprotein (SPE). (**A**,**B**). Effect of SPE on HaCaT cell viability. The MTT assay results showed that SPE did not exhibit cytotoxicity up to a concentration of 100 µg/mL. Data (n = 5) are presented as the mean ± SD. Means with different letters (a,b) indicate significant differences (*p* < 0.05, one-way ANOVA, Duncan’s Multiple Range test).

Amino acid composition and molecular weight measurements of sesame glycoprotein (SPE) were conducted using amino acid analyzer and MALDI-TOF/MS, as shown in Table 1 and Figure 1A. The molecular weight distribution of sesame glycoproteins displayed a relatively low molecular weight range from 263 to 310 Da, with an average molecular weight of 263.8 Da. This indicates that the sesame glycoprotein predominantly consists of small glycopeptide fragments. In comparison to larger cosmetic peptides, which typically have a molecular weight below 2000 Da to ensure skin absorption, the sesame glycoproteins fall well below this threshold, suggesting they can be efficiently absorbed through the skin [22]. Therefore, when used as a cosmetic material, sesame glycoproteins are expected to contribute significantly to skin hydration and moisture retention.

Cell viability assays were performed on HaCaT keratinocytes following UV exposure (25 kJ/m^2^), with treatments of SPE and HPE at concentrations ranging from 5 to 80 μg/mL. As shown in Figure 1B, neither SPE nor HPE exhibited significant protective effects against UV-induced cytotoxicity, as no meaningful improvements in cell viability were observed in any of the treatment groups.

### 2.2. Inhibition of UV-Induced MMP-1 Protein Expression by SPE

**Figure 2:**
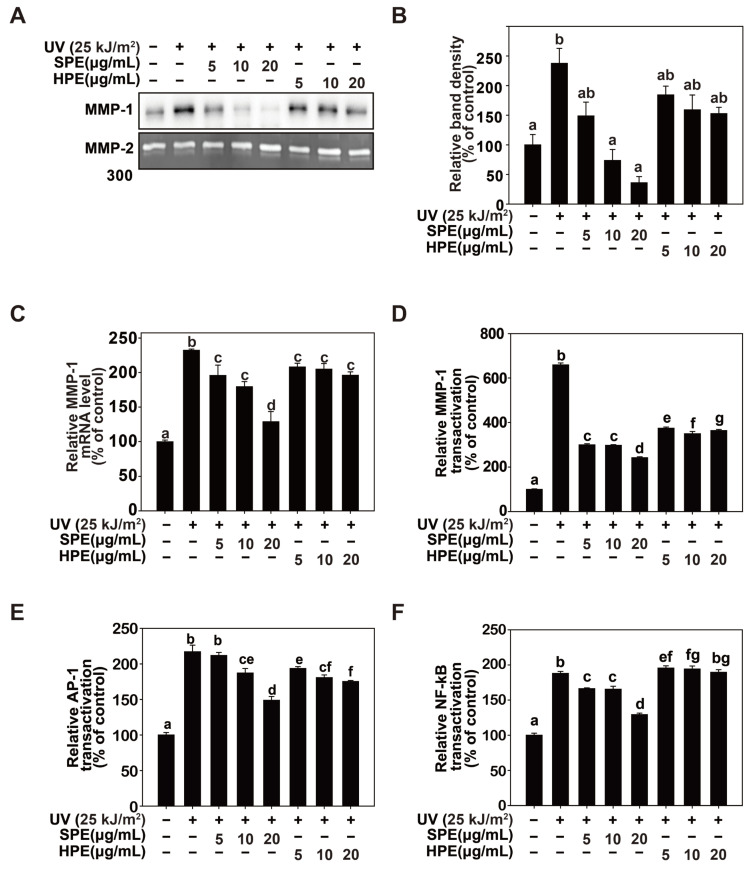
Effect of glycoproteins from sesame cake (SPE) on ultraviolet (UV)-induced matrix metalloproteinase (MMP)-1 expression. (**A**). HaCaT cells were seeded and incubated to 80% confluence. The cells were starved for 24 h, pretreated with SPE and hydrolyzed bovine collagen peptide Extract (HPE) at the indicated concentrations for 1 h, and then irradiated with 25 kJ/m^2^ UV. The cells were incubated for 48 h at 37 °C, and the medium was prepared for western blotting. MMP-2 was used as a loading control. Bands were quantified using NIH ImageJ software(version 1.52a). Data (n = 3) are presented as the mean ± SD. (**B**). Effects of SPE on UV-induced MMP-1 gene transcription in HaCaT cells. MMP-1 mRNA levels were analyzed using real-time quantitative PCR. Cells were pretreated with SPE at indicated concentrations for 1 h and then further treated with 25 kJ/m^2^ UV at 37 °C for 48 h. (**C**). Effects of SPE on UV-induced MMP-1 promoter activity. HaCaT cells transduced with MMP-1 promoter reporter plasmids were prepared, and MMP-1 promoter activity was measured as described in Section 4. (**D**). HaCaT cells transduced with MMP-1 reporter plasmids were treated with the indicated concentrations of HPE and SPE and exposed to UV (25 kJ/m^2^). (**E**,**F**) AP-1 and NF-κB transactivation were measured using a luciferase reporter gene assay. Data (n = 5) represent the mean ± SD. Means with different letters (a–g) indicate significant differences (*p* < 0.05, one-way ANOVA, Duncan’s Multiple Range test).

Evaluating the effects of sesame glycoproteins (SPE), a comparative study was conducted using hydrolyzed bovine collagen peptide extract (HPE), a well-known peptide for improving skin health. Bovine collagen was included as a negative control to assess the efficacy of SPE in preventing UV-induced skin damage. This study demonstrated that SPE significantly inhibited UV-induced MMP-1 expression at both the protein (Figure 2A) and mRNA levels (Figure 2B) without inducing cytotoxicity (Figure 1B). Western blotting revealed that SPE-treated cells exhibited a marked reduction in MMP-1 protein levels upon UV exposure, whereas HPE-treated cells exhibited only a moderate decrease. Similarly, quantitative real-time PCR (qRT-PCR) analysis confirmed that SPE effectively suppressed UV-induced MMP-1 mRNA expression. The mRNA levels of MMP-1 were significantly lower in SPE-treated cells than in HPE-treated cells, indicating that SPE exerted its inhibitory effects at the transcriptional level. Further mechanistic studies using luciferase reporter assays provided insights into the transcriptional regulation of MMP-1 by SPE (Figure 2C). Cells transfected with an MMP-1 promoter-driven luciferase reporter construct and treated with SPE showed a substantial decrease in luciferase activity following UV exposure. Additionally, luciferase reporter assays for AP-1 and NF-κB, major transcription factors involved in MMP-1 expression, revealed that SPE effectively inhibits their activity. UV-induced activation of AP-1 (Figure 2E) and NF-κB (Figure 2F) was significantly reduced in SPE-treated cells compared to that in HPE-treated cells. These findings collectively indicate that SPE has a potent inhibitory effect on UV-induced MMP-1 expression at both the transcriptional and translational levels without inducing cytotoxicity. The use of reporter gene assays further supported the role of SPE in modulating key transcription factors involved in MMP-1 regulation.

### 2.3. Inhibition of UV-Induced Phosphorylation of AKT and MAPK Pathway by SPE

**Figure 3:**
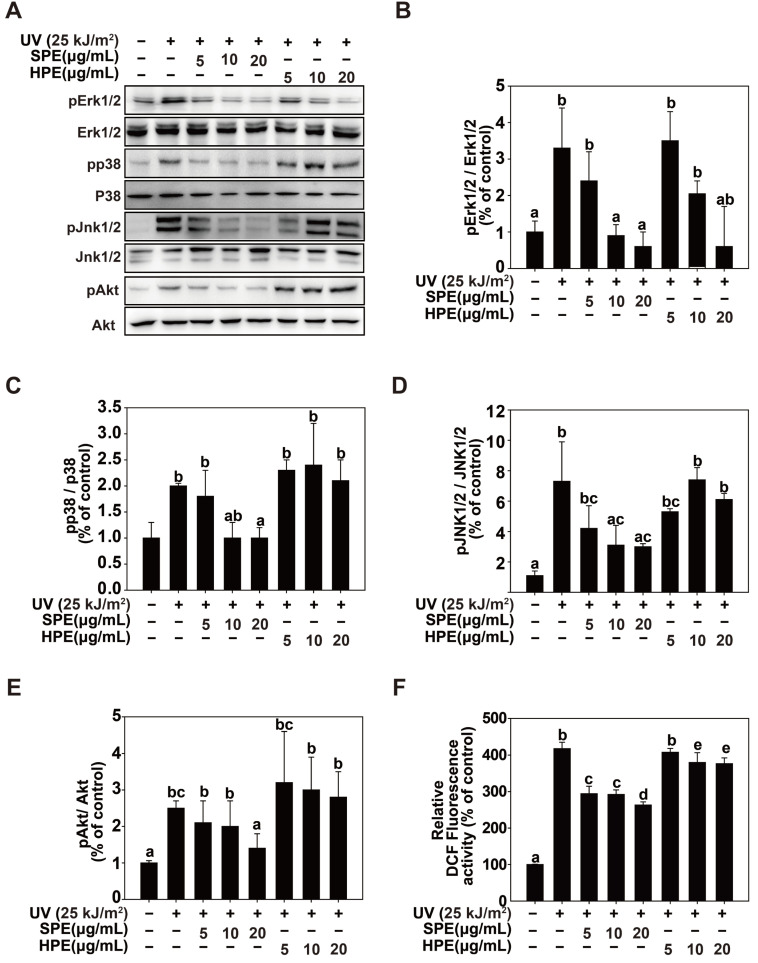
Effects of HPE and glycoproteins from sesame cake (SPE) on UV-induced MAPK, Akt activation, and reactive oxygen species (ROS) production in HaCaT cells. (**A**–**E**). Phosphorylation and total protein levels of MAPK and Akt pathways were analyzed by Western blotting. HaCaT cells were treated with different concentrations of HPE and SPE (5, 10, and 20 µg/mL) and exposed to UV (25 kJ/m^2^). The relative band strength of phosphorylated Erk1/2, Jnk1/2, p38, and Akt compared to total proteins was quantified using ImageJ software (V.1.52a). Relative fold changes compared to the control are indicated above each band. Data from (**A**) were quantified and presented in (**B**–**E**) for pErk1/2, pJnk1/2, pp38, and pAkt, respectively. (**F**). Effect of HPE and SPE on UV-induced ROS production. HaCaT cells were treated with HPE and SPE (5, 10, and 20 µg/mL) and then exposed to UV radiation (25 kJ/m^2^). ROS production was measured using the DCF fluorescence assay. Data represent the mean ± SD (n = 5). Different letters (a–e) indicate significant differences (*p* < 0.05, one-way ANOVA, Duncan’s multiple range test).

AKT is a major player in the PI3K/AKT signaling pathway, which is essential for cell survival and proliferation. We found that SPE treatment markedly reduced the levels of pAKT in UV-exposed cells, suggesting that SPE can interfere with the activation of this pathway, thereby potentially reducing the cell survival signals that contribute to UV-induced damage. Similarly, ERK is a key event in the MAPK/ERK pathway that regulates cell division and differentiation. The inhibition of pERK by SPE indicates that SPE might impair the ability of cells to proliferate and respond to UV-induced stress (Figure 3). This could be beneficial for preventing the overactivation of cellular mechanisms that lead to MMP-1 production and subsequent tissue damage. pp38 and pJNK are critical components of stress-activated protein kinase pathways that respond to various stress stimuli, including UV radiation. These pathways are involved in regulating inflammatory responses and apoptosis. This study demonstrated that SPE significantly reduced the phosphorylation of both p38 and JNK, suggesting that SPE could mitigate the cellular stress response and inflammation induced by UV exposure.

### 2.4. Antioxidant Capacity of SPE Compared to HPE

The antioxidant capacity of SPE and HPE was evaluated using two different assays: the VCEAC and tannic acid equivalent (TAE) assays. The VCEAC assay results indicated that SPE has a significantly higher antioxidant capacity compared to HP, with SPE showing a value of 6.78 ± 0.37 mg VCEAC/g, whereas HPE showed only 1.04 ± 0.35 mg VCEAC/g. This suggests that the antioxidant capacity of SPE was approximately 6.5-fold that of HPE when measured in terms of vitamin C equivalents. Similarly, the TAE assay results revealed that the antioxidant capacity of SPE is considerably higher compared to that of HPE, with SPE showing a value of 23.13 ± 0.27 mg TAE/g, whereas HPE showed only 4.62 ± 0.15 mg TAE/g. This indicates that SPE’s antioxidant capacity is approximately five times greater than that of HPE when measured in terms of TAE (Table 2). The inhibitory effect of SPE on the phosphorylation of these signaling molecules is attributed to its ability to reduce UV-induced ROS generation. ROS are highly reactive molecules that can cause significant cellular damage and play a crucial role in the activation of signaling pathways [23]. UV radiation increases ROS production, leading to the activation of signaling molecules, such as AKT, ERK, p38, and JNK, which in turn mediate cellular responses to stress and contribute to the upregulation of MMP-1. Using the DCFDA assay, we observed an increase in intracellular ROS levels in the UV-treated HaCaT cells. The elevated ROS levels were effectively inhibited by SPE but not by HPE (Figure 3F). These results suggest that SPE inhibits MMP-1 activity by reducing ROS levels, thereby interfering with the signaling pathways.

### 2.5. Effects of SPE on UV-Induced Skin Wrinkles in SKH-1 Hairless Mice

**Figure 4:**
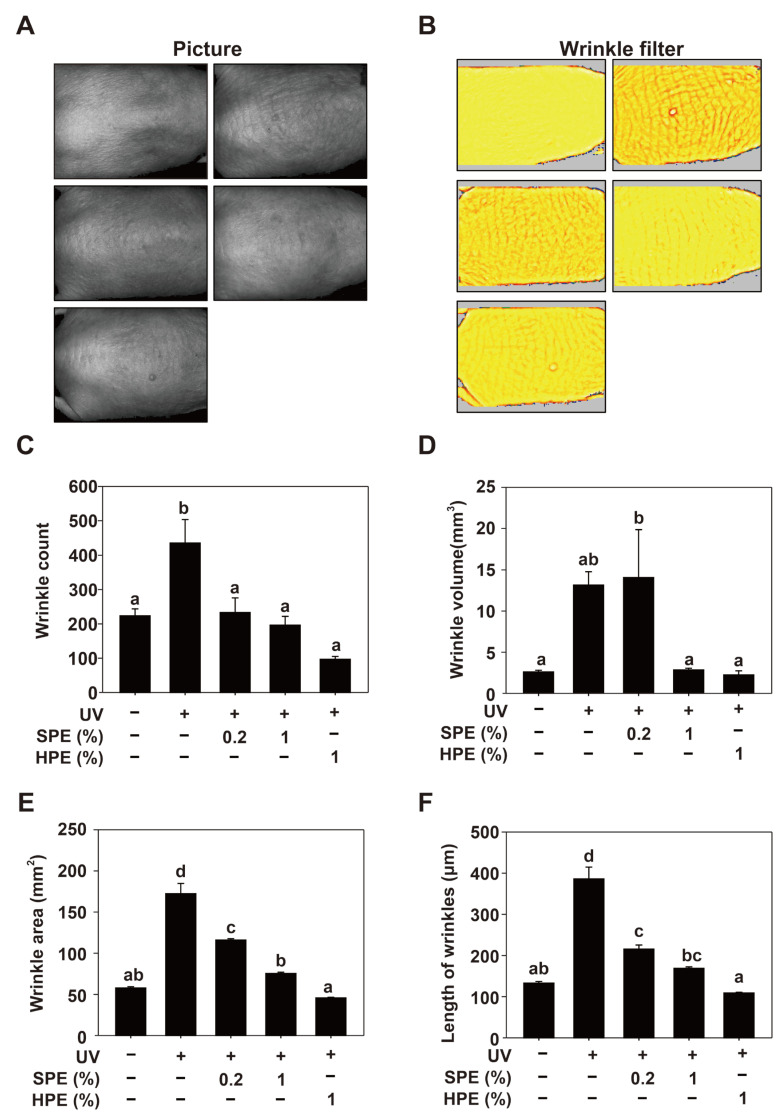
Effects of HPE and glycoproteins from sesame cake (SPE) on UV-induced wrinkle formation in SKH-1 hairless mice. (**A**). Representative images of the dorsal skin of SKH-1 hairless mice from different treatment groups. Mice were exposed to UV radiation and treated with different concentrations of HPE and SPE (0.2%, 0.5%, and 1%). (**B**). Pseudocolor images of the wrinkle analysis showing the depth and area of wrinkles. (**C**–**F**). Quantitative analysis of wrinkle formation in the different treatment groups: (**C**). Average wrinkle length (μm), (**D**). Wrinkle area (mm^2^), (**E**). Wrinkle volume (mm^3^), and (**F**). Wrinkle count. Mice (n = 8 per group) were treated with different concentrations of HPE and SPE (0.2%, 0.5%, and 1%) and exposed to UV. Detailed experimental procedures are described in the Section 4. Data are presented as mean ± SE. Means with different letters (a–d) indicate significant differences (*p* < 0.05, one-way ANOVA, Duncan’s Multiple Range test).

This study demonstrates that SPE effectively reduces UV-induced skin wrinkles in SKH-1 hairless mice. Visual assessment of the skin surface showed that mice treated with SPE exhibited significantly fewer and less severe wrinkles than those treated with HPE or UV alone. The 0.5% SPE group displayed the most noticeable reduction in wrinkle formation (Figure 4A,B). The quantitative analysis of wrinkle parameters further supported these findings. The SPE-treated groups, particularly at 0.5% concentration, showed significant reductions in wrinkle count, length (Figure 4C), volume (Figure 4D), area (Figure 4E), and length (Figure 4F) compared with the UV group. The 0.5% SPE group had the smallest wrinkle area and volume and the lowest wrinkle count, indicating the highest efficacy in mitigating UV-induced skin damage. In contrast, the HPE-treated groups showed moderate improvements but were less effective than the SPE-treated groups. These results suggest that SPE is a potent agent for reducing UV-induced skin wrinkles, highlighting its potential for skincare applications.

### 2.6. Effects of SPE on UV-Induced Histological Changes in SKH-1 Hairless Mice

**Figure 5:**
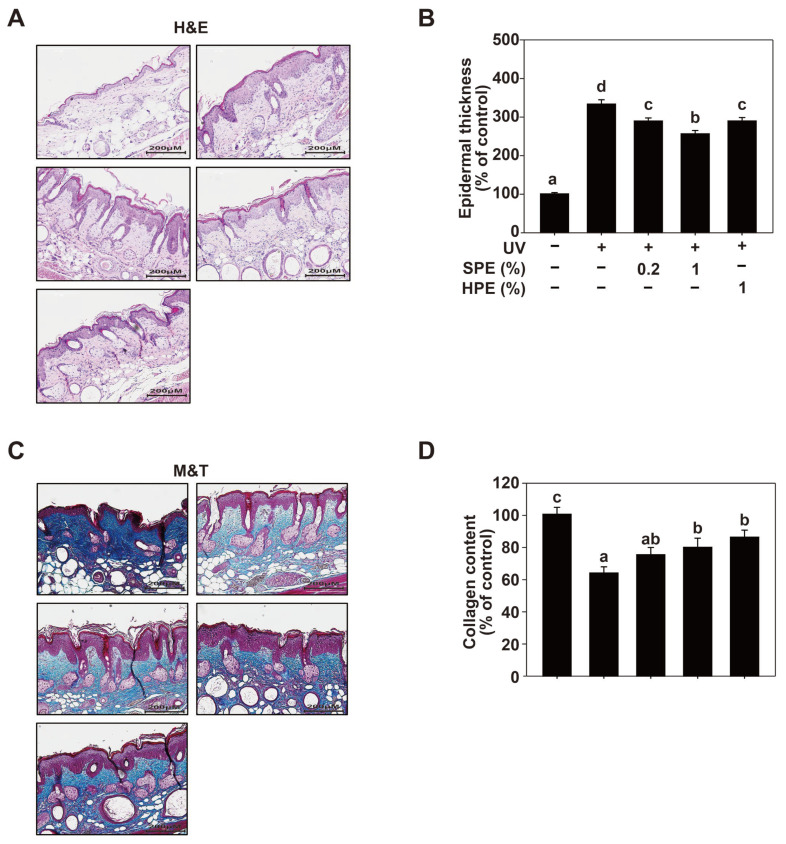
Histological analysis of the effect of HPE and SPE on UV-induced skin damage in SKH-1 hairless mice. (**A**,**B**). Representative H&E-stained sections of the dorsal skin from different treatment groups. The mice were exposed to UV radiation and treated with various concentrations of HPE and SPE (0.2%, 0.5%, and 1%). (**C**,**D**). Representative Masson’s trichrome-stained sections showing collagen content in the dermis. The mice (n = 8 per group) were treated with different concentrations of HPE and SPE (0.2%, 0.5%, and 1%) and exposed to UV. The detailed experimental procedures are described in Section 4. Data are presented as the mean ± SE. Means with different letters (a–d) are significantly different (*p* < 0.05, one-way ANOVA, Duncan’s Multiple Range test).

Hematoxylin and eosin (H&E) staining was performed to assess epidermal thickness (Figure 5A,B). The UV group showed a significant increase in epidermal thickness compared with the control group, indicating skin damage due to UV exposure. Treatment with SPE, particularly at 0.5% concentration, significantly reduced the UV-induced increase in epidermal thickness. In contrast, the 0.5% HPE group showed a moderate reduction in epidermal thickness compared with the UV group. The bar graph on the right quantifies epidermal thickness as a percentage of the control. The UV group exhibited the highest epidermal thickness. The SPE-treated groups, particularly the 0.5% SPE group, showed a significant reduction in epidermal thickness, bringing it closer to the control levels. The 0.5% HPE group showed less reduction than the 0.5% SPE group, indicating lower efficacy. Masson’s trichrome (MT) staining was used to evaluate the collagen content in the skin (Figure 5C,D). The UV group displayed a significant reduction in collagen content compared with the control group, indicating UV-induced collagen degradation. Treatment with SPE, particularly at 0.5%, significantly increased the collagen content compared to that in the UV group, suggesting a protective effect against collagen degradation. The 0.5% HPE group showed a moderate increase in collagen content compared to the UV group. The bar graph on the right quantifies collagen content as a percentage of the control. The UV group showed the lowest collagen content. The SPE-treated groups, especially the 0.5% SPE group, showed a significant increase in collagen content, which was restored to the control levels. The 0.5% HPE group exhibited a smaller increase in collagen content than the SPE-treated groups, indicating a lower protective effect.

## 3. Discussion

This study highlights the potential of SPE as a natural agent to mitigate UV-induced skin damage, particularly in the context of photoaging. The data presented elucidate the multifaceted protective effects of SPE, which not only include the inhibition of MMP-1 expression but also the modulation of key signaling pathways and the preservation of skin histology [24]. These findings offer promising insights into the development of advanced skincare formulations that leverage the bioactive properties of SPE.

A key finding of this study is the significant inhibition of UV-induced MMP-1 expression by SPE treatment. MMP-1 is a collagenase that degrades collagen, a critical component of the extracellular matrix of the skin [4]. Its upregulation upon UV exposure primarily contributes to photoaging, leading to wrinkle formation and loss of skin elasticity [1]. The ability of SPE to markedly reduce MMP-1 levels suggests its potential to preserve collagen integrity and prevent structural deterioration associated with UV-induced aging [25]. This effect is likely mediated through the downregulation of AP-1 and NF-κB, the transcription factors that play crucial roles in MMP-1 induction [2]. The inhibition of these factors by SPE highlighted its potential to intervene at the transcriptional level and prevent collagen degradation.

This study also highlights the inhibitory effects of SPE on the phosphorylation of key signaling molecules such as AKT, ERK, p38, and JNK, which are activated by UV-induced oxidative stress [2]. These signaling pathways are involved in cellular responses such as proliferation, inflammation, and apoptosis. The reduction in the phosphorylation levels of these molecules by SPE indicates its ability to modulate these pathways, thereby reducing cellular stress and inflammatory responses triggered by UV exposure [4]. This modulation likely contributes to reduced MMP-1 expression, as these pathways are intricately linked to MMP regulation [26].

Another important aspect of the protective mechanism of SPE is its high antioxidant capacity. Comparative analysis using VCEAC and TAE assays demonstrated that SPE possesses substantially higher antioxidant activity than HPE [27]. Antioxidants play a crucial role in neutralizing ROS, which are abundantly generated upon UV exposure and cause oxidative damage to cellular components [28]. By effectively reducing ROS levels, SPE not only mitigates direct oxidative damage but also prevents the activation of ROS-mediated signaling pathways [29]. The dual action of the direct antioxidant activity and pathway modulation underscores the protective role of SPE against UV-induced skin damage.

In vivo experiments using SKH-1 hairless mice further validated the protective effects of SPE against UV-induced skin damage [30,31,32]. The significant reduction in wrinkle formation, as evidenced by the decreased length, area, volume, and wrinkle count in the SPE-treated groups, highlights its efficacy in preventing the visible signs of photoaging. Histological analyses provided additional support, showing that SPE treatment effectively mitigated the UV-induced increases in epidermal thickness and collagen degradation. These findings suggest that the SPE prevents functional damage and preserves the structural integrity of the skin.

This study also highlights the differential efficacy of SPE compared to HPE, with SPE consistently showing superior protective effects. This highlights the unique bioactive properties of sesame-derived compounds, particularly the glycoproteins and antioxidants enriched in SPE, which contribute to their enhanced efficacy.

In conclusion, this study demonstrated that SPE is a potent natural agent for protecting the skin against UV-induced damage. Its ability to inhibit MMP-1 expression, modulate critical signaling pathways, and provide robust antioxidant protection makes it a valuable component in anti-aging skincare formulations. These findings pave the way for further research on the molecular mechanisms underlying the protective effects of SPE and its potential long-term benefits in humans. Future studies should focus on clinical trials to validate these findings in humans and explore the formulation of SPE in various skincare products to maximize its protective effects against photoaging.

## 4. Materials and Methods

### 4.1. Reagents

High Dulbecco’s modified Eagle medium (DMEM), penicillin–streptomycin solution, and trypsin–EDTA solution were purchased from Welgene (Gyeongsan, Republic of Korea). Fetal bovine serum (FBS) was obtained from Atlas Biologicals (Fort Collins, CO, USA). L-ascorbic acid was acquired from LPS Solution (Daejeon, Republic of Korea). Tannic acid and Folin’s phenol reagent were purchased from Sigma-Aldrich (St. Louis, MO, USA). MMP-1 antibody was procured from R&D Systems, Inc. (Minneapolis, MN, USA). Phosphorylated extracellular signal-regulated kinase (ERK) 1/2, total ERK1/2, total Akt, total c-Jun N-terminal kinase 1 (JNK1), MKK3/6, and MEK1/2 were obtained from Santa Cruz Biotechnology (Santa Cruz, CA, USA). ECL Prime Western Blotting Detection Reagent was purchased from Amersham (Little Chalfont, UK).

### 4.2. SPE and HPE Preparation

SPE and HPE was provided by CNA Biotech (Cheongju, Republic of Korea). We extracted glycoprotein by referencing our previous studies [33,34]. The sesame cake was cleansed with distilled water to remove impurities and then blended with distilled water in a 1:4 ratio using a chopper to achieve a particle size of 10–100 μm. To this mixture, 0.3–0.5% of organic acids such as acetic acid and proteolytic enzymes including trypsin and papain (0.1–0.5 w%) were added per 1 kg of ground material. The solution was then hydrolyzed at 45–55 °C for 24 h. The hydrolyzed solution was centrifuged and filtered at 78–85 °C to inactivate the enzymes, yielding the hydrolyzed glycoprotein. This was subsequently freeze-dried to produce the SPE powder. The bovine gelatin was mixed with distilled water at a 5:7.5 ratio and combined with alcalase at 0.5% (*w*/*w*) of the gelatin weight. The mixture was then hydrolyzed at 55–60 °C for 4–5 h using enzymatic hydrolysis. After hydrolysis, the bovine gelatin solution was filtered using filter paper and cloth, and then sterilized at 121 °C for at least 30 s. The final hydrolyzed peptide extract was obtained by spray drying the solution to produce the HPE powder.

### 4.3. Amino Acid Composition Analysis and Molecular Weight Determination of SPE

The amino acid composition of SPE was analyzed using a Waters 510 HPLC equipped with an automated amino acid analyzer (Waters Pico Tag HPLC system, Milford, MA, USA) following PITC derivatization. For extraction and sample preparation, 1000 μL of the sample was hydrolyzed and derivatized using the PICO tag method. After derivatization, 20 μL of the prepared sample was taken from the total 400 μL and loaded onto the HPLC for chromatographic analysis. The molecular weight of SPE was determined using a MALDI-TOF/TOF™ 5800 system (AB SCIEX, Framingham, NY, USA) operated in MS reflector mode (positive ion mode). The matrix used was α-cyano-4-hydroxycinnamic acid at a concentration of 5 mg/mL, prepared in a solution of 0.1% trifluoroacetic acid (TFA) and 50% acetonitrile (ACN). Calibration of the instrument was carried out using a mixture of peptides, including Arg^1^-bradykinin (904.468 Da), angiotensin I (1296.685 Da), ACTH (1–17) (2093.087 Da), ACTH (18–39) (2465.199 Da), and ACTH (7–38) (3657.9294 Da). Data processing involved baseline correction and smoothing of the spectra using a Gaussian smooth filter (5 points). The software used for analysis was TOF/TOF™ Series Explorer (version 4.1.0).

### 4.4. 2,2′-Azinobis (3-Ethylbenzothiazoline-6-sulfonic acid) Assay

The 3-Ethylbenzothiazoline-6-sulfonic acid (ABTS) radical cation decolorization assay was performed to measure the antioxidant capacity of the samples [35]. The ABTS radical cation (ABTS•⁺) was generated by reacting 7 mM ABTS stock solution with 2.45 mM potassium persulfate (K_2_S_2_O_8_) and allowing the mixture to stand in the dark at room temperature for 12–16 h before use. To prepare the ABTS•⁺ solution, a 7 mM ABTS solution was prepared by dissolving ABTS in distilled water. A 2.45 mM potassium persulfate solution was prepared by dissolving K_2_S_2_O_8_ in distilled water. The ABTS stock solution was mixed with the potassium persulfate solution in a 1:1 ratio and stored in the dark at room temperature for 12–16 h to form ABTS•⁺. The samples were diluted in the appropriate solvents to achieve the required concentrations for the assay. For the assay, the ABTS•⁺ solution was diluted with phosphate-buffered saline (PBS) to yield an absorbance of 0.70 ± 0.02 at 734 nm. Subsequently, 20 µL of the diluted sample or standard antioxidant solution (vitamin C) was mixed with 980 µL of the diluted ABTS•⁺ solution. The mixture was incubated at room temperature for 6 min and the absorbance was measured at 734 nm using a spectrophotometer. The antioxidant capacities of the samples were expressed as vitamin C equivalent antioxidant capacity (VCEAC). Percentage inhibition of the absorbance as measured at 734 nm. All experiments were performed in triplicate, and the results were expressed as the mean ± standard deviation (SD).

### 4.5. Total Phenolic Contents

A total of 100 μL of distilled water was mixed with 10 μL of Folin reagent and incubated in the dark for 5 min. Subsequently, 100 μL of 7% Na_2_CO_3_ solution was added and incubated in the dark for an additional 30 min [27]. The O.D. at 750 nm should fall between 0.5 and 0.7. The absorbance was then inputted into a standard curve to calculate the comparative quantitative value (*w*/*v*) and divided by the sample concentration (*w*/*v*) to measure the total phenolic content (*w*/*v*).

### 4.6. Total Flavonoid Contents

A total of 25 μL of the sample was mixed with 125 μL of distilled water and 40 μL of 5% NaNO_2_ and incubated in the dark for 5 min [27]. Subsequently, 55 μL of 10% AlCl_3_ was added and incubated for another 5 min, followed by the addition of 50 μL of 1 N NaOH and an additional 30-minute incubation in the dark. Measurements were performed at a wavelength of 510 nm. The absorbance was plotted on a standard curve to calculate the comparative quantitative value (*w*/*v*), and the concentration was used to measure the total flavonoid content (quercetin equivalent, mg QE/g).

### 4.7. Cell Culture and UV Light Exposure

HaCaT cells were purchased from CLS Cell Lines Services GmbH (Heidelberg, Germany) and cultured in DMEM supplemented with FBS and 1% penicillin/streptomycin solution at 37 °C in a 5% CO_2_ incubator. Cells were exposed to simulated UV (UV) light, comprising approximately 95% UVA (315–400 nm) and 5% UV (280–315 nm) wavelengths, using a lamp designed to mimic solar UV light (Q-Lab Corporation, Westlake, OH, USA) at a dose of 25 kJ/m^2^ in serum-free DMEM.

### 4.8. Cell Viability Assay

Cell viability was assessed using a 3-[4,5-dimethylthiazol-2-yl]-2,5-diphenyltetrazolium bromide (MTT) assay. Cells were seeded at a density of 2 × 10^4^ cells/mL in a 96-well cell culture plate using DMEM supplemented with 10% FBS and 1% penicillin/streptomycin and incubated at 37 °C in a 5% CO_2_ incubator for 24 h. Once confluency exceeded 80%, the cells were washed twice with serum-free DMEM and incubated in serum-free medium for another 24 h. After incubation, the cells were washed twice with serum-free medium and exposed to various doses of UV (6.25, 12.5, 25, 50, and 100 mJ/cm^2^) for 1 h. After UV exposure, cells were incubated for 24, 48, and 72 h. Subsequently, MTT solution (0.45 mg/mL) was added and incubated for 2 h at 37 °C in a 5% CO_2_ incubator. The supernatant was then removed, and DMSO (200 μL) was added. Absorbance was measured at 570 nm using a microplate reader (BioTek, Winooski, VT, USA).

### 4.9. 2′,7′-Dichlorodihydrofluorescein Diacetate Assay

To measure intracellular reactive oxygen species (ROS) production following UV exposure, HaCaT cells were seeded at a density of 2 × 10^4^ cells/mL in a 96-well black cell culture plate and incubated at 37 °C in a 5% CO_2_ incubator for 24 h. After confirming > 80% confluence, the cells were washed twice with serum-free DMEM and incubated for 24 h in serum-free medium. Cells were then washed twice with Hank’s balanced salt solution (HBSS) and incubated with 2′,7′-dichlorofluorescin diacetate (DCF-DA) at a final concentration of 25 μM/mL for 30 min. Following another two washes with HBSS, SPE and HPE were prepared at different concentrations (5, 10, and 20 μg/100 μL) and applied to the cells for 1 h before UV exposure (25 mJ/cm^2^). The generation of ROS was measured 2 h post-exposure using a Cell Imaging Multi-Mode Reader (BioTek, Winooski, VT, USA) at a wavelength of 485–530 nm ± 20 nm.

### 4.10. Luciferase Reporter Gene Assay

HEK293T cells were transfected with pGF-AP-1-mCMV-EF1-Puro, pGF-NF-κB-mCMV-EF1-Puro, and pGF-MMP-1-mCMV-EF1-Puro vectors along with packaging vectors (psPAX and pMD2.0G) using jetPEI according to the manufacturer’s instructions. The medium was changed 24 h post-transfection, and the cells were further incubated for 36 h. Viral particles were produced using a syringe filter (0.45 μm). HaCaT cells were infected overnight with 8 μg/mL polybrene (EMD Millipore, Burlington, MA, USA). After replacing the culture medium with fresh medium, the cells were incubated for 24 h and selected using 2 mg/mL puromycin (Sigma, Saint Louis, MO, USA) for 36 h. After incubation in a serum-free medium for 24 h, the cells were treated with SPE for 1 h before UV exposure. Transactivation was measured 12 (AP-1) or 24 h (NF-κB, MMP-1) later using the Luciferase Reporter Gene Analysis Kit (Promega, Madison, WI, USA).

### 4.11. Western Blot

Cells were prepared and treated in a manner similar to that for the DCF-DA assay. After treatment and UV exposure, cells were collected, washed with PBS, and lysed using a buffer containing 50 mM Tris-HCl (pH 8.0), 0.15 M NaCl, 1% NP-40, 0.1% SDS, 0.5% deoxycholate, 1 mM dithiothreitol, 1 mM phenylmethylsulfonyl fluoride, and 1 mM Na_3_VO_4_. The lysates were then centrifuged at 13,000 rpm, and the supernatant was transferred to fresh microtubes. Protein concentrations were determined using the D/C Protein Assay Kit (Bio-Rad, Hercules, CA, USA). Equal amounts of proteins were separated using SDS-PAGE on a 10% polyacrylamide gel and transferred onto a PVDF membrane. The membrane was blocked in 5% non-fat milk for 2 h and incubated with primary antibodies overnight at 4 °C. After washing thrice with TBS-T, the membrane was incubated with HRP-conjugated secondary antibodies diluted in 5% skim milk for 3 h at 4 °C. After three additional washes with TBS-T, the membrane was treated with ECL and imaged using a FUSION Solo S system (Vilber Lourmat, Paris, France).

### 4.12. Gelatin Zymography

To analyze the activity of matrix metalloproteinases (MMPs), specifically MMP-2, after treatment and UV exposure, cell culture supernatants were collected and centrifuged at 13,000 rpm. The protein concentration in the supernatant was normalized using a D/C Protein Assay Kit (Biorad, Hercules, CA, USA). The samples were mixed with 6× zymogram buffer and analyzed by SDS-PAGE containing 1% gelatin and 10% polyacrylamide. The gel was then washed twice for 20 min in renaturing buffer followed by a wash in developing buffer for 20 min before incubation at 37 °C for 48 h on a shaker. The gel was stained with Coomassie brilliant blue for 1 h and then destained with destaining buffer until clear bands indicating MMP activity were observed. The results were documented using a FUSION Solo S system (V.070, Vilber Lourmat, Marne-la-Vallée, France).

### 4.13. Animal Experiments

The research utilized 6-week-old female SKH-1 hairless mice weighing 20–22 g were obtained from Central Laboratory Animal Inc., Seoul, Korea. These mice were housed in a temperature-controlled room set at 23 ± 2 °C with a 12-hour light/dark cycle, with *ad libitum* access to food and water. The study design was approved by the Ethics Committee and adhered to the guidelines for animal care and use established by the Korea National University of Transportation (KNUTIACUC 2023-2). The mice were randomly divided into five groups (each, n = 8): (1) control (mice fed a normal diet without any treatment); (2) UV (mice fed a normal diet and exposed to UV radiation); (3) 0.1% SPE (mice exposed to UV radiation and fed a diet with 0.1% (*w*/*w*) SPE); (4) 0.5% SPE (mice exposed to UV radiation and fed a diet with 0.5% (*w*/*w*) SPE); and (5) 0.5% HPE (mice exposed to UV radiation and fed a diet with 0.5% (*w*/*w*) HPE). For UV irradiation, the dorsal regions of mice were exposed to UV radiation three times weekly. The exposure intensity was gradually increased by 1 minimal erythema dose (MED = 0.5 kJ/m^2^) each week until it reached 4 MED, which was maintained for 16 weeks using a BLX312 UV crosslinker (Vilber Lourmat, Marne-la-Vallée, France). The water content within the dorsal skin of mice was evaluated using skin capacitance measurements under controlled humidity and temperature conditions on the day before euthanasia. Wrinkle formation was assessed 16 weeks after UV irradiation using a skin wrinkle-measuring device (Primos CR; Canfield Scientific, Parsippany, NJ, USA).

### 4.14. Histological Analysis

Skin tissue samples were collected from the dorsal regions of the mice immediately after euthanasia. The tissues were fixed in 10% neutral-buffered formalin for 24 h, dehydrated through a graded series of ethanol, cleared in xylene, and embedded in paraffin wax. Serial sections of 5 μm thickness were cut using a microtome (Leica RM2235, Leica Microsystems, Wetzlar, Germany) and mounted on glass slides. For general histological evaluation, sections were stained with hematoxylin and eosin (H&E). To assess collagen fiber deposition, Masson’s trichrome staining was performed following standard protocols. Stained sections were examined and digital images were captured using the Cytation 5 imaging system (BioTek Instruments, Winooski, VT, USA). Quantitative analysis of epidermal and dermal thickness, as well as collagen fiber density, was conducted using the dedicated Gen5 software(V.3) associated with the Cytation 5 system.

### 4.15. Statistical Analysis

Results were presented as the mean ± standard deviation (SD) of three independent experiments. Data were analyzed using the SPSS software (version 21.0; IBM, Armonk, NY, USA). Differences between groups were assessed using analysis of variance (ANOVA), followed by Duncan’s post hoc test when appropriate. Statistical significance was set at *p* < 0.05.

## Figures and Tables

**Table 1:** Amino acid composition of sesame glycoproteins (SPE). The sample was analyzed using the method described in the Materials and Methods Section 4.

Amino Acid	%
Asp	10.9
Glu	16.4
OH-Pro	0.1
Ser	9.4
Gly	19.2
His	1.6
Arg	6.4
Thr	3.2
Ala	10.8
Pro	3.1
Tyr	2.0
Val	3.8
Met	0.8
Ile	2.4
Leu	4.1
Phe	2.2
Lys	3.2

**Table 2:** Comparison of Antioxidant and Phenolic Contents between SPE and HPE. Total antioxidant activities of the SPE and HPE extracts are presented. The values were estimated as vitamin C equivalent antioxidant capacity (VCEAC) per gram of dry weight. Total phenolic content of SPE vs. HPE. Values are mg of gallic acid equivalents (GAE) per gram of dry weight. Total flavonoid content in SPE and HPE extracts are presented. Quercetin equivalent (QE) per g of dry weight. The quantitative values are presented.

Method Comparison		Total ABTs Contents Vitamin C Equivalents	Total Phenolic Contents Tannic Acid Equivalents	Total Flavonoid Contents Quercetin Equivalents
Unit		mg VCEAC/g	mg GAE/g	mg QE/g
Value	SPE	6.78 ± 0.37	23.13 ± 0.27	77.32 ± 0.48
	HPE	1.04 ± 0.35	4.62 ± 0.15	0.27 ± 0.03

## Data Availability

Data are contained within the article.
